# Grey matter changes can improve the prediction of schizophrenia in subjects at high risk

**DOI:** 10.1186/1741-7015-4-29

**Published:** 2006-12-07

**Authors:** Dominic E Job, Heather C Whalley, Andrew M McIntosh, David GC Owens, Eve C Johnstone, Stephen M Lawrie

**Affiliations:** 1 Division of Psychiatry, School of Molecular and Clinical Medicine, The University of Edinburgh, The Royal Edinburgh Hospital, Morningside Park, Edinburgh, Scotland, EH10 5HF, UK

## Abstract

**Background:**

We hypothesised that subjects at familial high risk of developing schizophrenia would have a reduction over time in grey matter, particularly in the temporal lobes, and that this reduction may predict schizophrenia better than clinical measurements.

**Methods:**

We analysed magnetic resonance images of 65 high-risk subjects from the Edinburgh High Risk Study sample who had two scans a mean of 1.52 years apart. Eight of these 65 subjects went on to develop schizophrenia an average of 2.3 years after their first scan.

**Results:**

Changes over time in the inferior temporal gyrus gave a 60% positive predictive value (likelihood ratio >10) of developing schizophrenia compared to the overall 13% risk in the cohort as a whole.

**Conclusion:**

Changes in grey matter could be used as part of a predictive test for schizophrenia in people at enhanced risk for familial reasons, particularly for positive predictive power, in combination with other clinical and cognitive predictive measures, several of which are strong negative predictors. However, because of the limited number of subjects, this test requires independent replication to confirm its validity.

## Background

In recent years it has become apparent that transient or partial psychotic symptoms occur often in the general population [1] and that such features are more common in those who go on to develop schizophrenia [2,3]. The Edinburgh High Risk Study has prospectively examined, over a period of 10 years, the mental state and the brain structure of more than 150 young people at enhanced risk of schizophrenia for familial reasons [3]. During that time 21 have developed schizophrenia and a further 60 have had transient, isolated or partial psychotic symptoms on at least one occasion but have not developed schizophrenia, and most have now passed the maximum age of risk. Clearly, only a minority of those with such symptoms will develop schizophrenia. Simple clinical and cognitive measures are remarkably effective in predicting those who will not develop schizophrenia [3], but these measures are not as good at predicting those predisposed subjects who will develop psychosis. In this report, we evaluate whether changes in brain structure over time [4] perform better than these predictive behavioural measures.

## Methods

In our previous work [4], we compared the changes over time in 65 high-risk subjects from the sample in the Edinburgh High Risk Study (34 males and 31 females, average age 21.4 (SD 2.7) years at first scan) who had two scans 1.76 (SD 0.45) years apart, at baseline and second scan. Fifty-seven of these people remain well, and eight had gone on to develop schizophrenia by June 2003, an average of 2.3 years (SD 1.0 years, range 0.6–3.4 years) after their first scan (7 of these 8 subjects were first scanned in 1995). No more of the remaining 57 well subjects had developed psychosis by January 2006. There were no significant differences in time between scans across the groups. The diagnosis of schizophrenia was made by PSE [Present State Examination]/Catego [5] and ICD-10 (10th revision of the *International Classification of Diseases*) criteria. Transient, isolated or partial psychotic symptoms fall within categories 2 and 3 of the modified PSE psychopathology classification described previously [6]. Gender distribution did not differ between those who are ill and those who remain well [3]. All subjects had serial, T1-weighted volumetric magnetic resonance imaging scans on the same 1.0 T Siemens scanner with a standard protocol (MPRAGE [magnetisation prepared rapid acquisition gradient echo]). Voxel-based morphometry (VBM) was performed using statistical parametric mapping methods – the SPM99 toolbox [7] – to map changes in grey matter over time [4]. Ethical approval was obtained from the Lothian Region Ethics Committee and all subjects gave written informed consent.

We previously hypothesised that the subjects who eventually developed schizophrenia would have reductions in grey matter, particularly in the temporal lobes, before developing the illness. A contrast was constructed, using a mask at voxel level, to examine changes in grey matter over time in those eight subjects who later developed schizophrenia, excluding any areas of change in high-risk subjects who had transient or isolated psychotic symptoms but did not develop schizophrenia. A detailed description of the previous analysis and imaging methods used were described earlier [4]. The results of our previous work showed that those subjects who went on to develop schizophrenia had more extensive reductions in the right cerebellum, left uncus and left inferior temporal gyrus than those who did not develop schizophrenia (*p*-corrected < 0.05).

Here, we extracted the change over time in grey matter densities for each subject at each of the three previously determined areas. Using the change in these density measures for each subject over the 18-month period, we tested multiple thresholds to determine the best cut-off point for a potential early diagnostic test. Given the current lack of any preventative treatments, we chose to balance false positives and false negatives. We give the data at obvious 'knee-points' on the receiver operator characteristic (ROC) curve.

## Results

### Predictive values

We used data from all 65 high-risk subjects as a basis for our test. The inferior temporal gyrus gave the best positive predictive value, 60%, with a negative predictive value of 92% (see Table 1). This threshold gave three true positives, two false positives, five false negatives and 55 true negatives. We were also interested in the possibility of prediction within those 18 subjects who had transient, isolated or partial psychotic symptoms; the findings are given in Table 2.

### Clinical usefulness

To determine the clinical usefulness of this VBM-based test, we need to know how it applies to an individual (familial high-risk subjects). For example, using the inferior temporal gyrus change as a test, the positive predictive value (60%) means that 60% of the subjects who test positive, i.e. for whom reductions in grey matter between the first and second scans lie above the threshold, will develop schizophrenia. The negative predictive value (92%) means that 92% of the subjects who test negative i.e. those who are below the threshold, will not develop schizophrenia. The likelihood ratio for a positive test (10.69) tells us that a subject with a positive test, i.e. a score above the threshold, is 10 times more likely to develop schizophrenia than a subject with a negative test. The negative likelihood ratio for this test (of 0.65) tells us that a subject who tests negative is nearly twice as likely to stay well as a subject with a positive test.

### Limitations

Due to the limited number of subjects, this test needs to be replicated with an independent sample to confirm its validity as a significant predictive measure. As this model was developed and tested on one sample, it is likely to be overestimated. Although we have a small number of subjects, it should be noted that these data are highly unusual, requiring 10 years of study to acquire. Lastly, it is not possible to predict schizophrenia in the general population using this test, as the prevalence of schizophrenia in the general population is approximately 1%.

## Discussion

Our previous volumetric region of interest (ROI) study [8-10], which included the subset of subjects used in this VBM study, measured the amygdalo-hippocampal complex and temporal lobe volumes, but not the cerebellum. These volumetric ROI measurements of changes over time were non-significant predictors of schizophrenia [11,3]. This is probably because manual volumetric ROI is more variable than VBM, particularly for small volumes, and volumetric ROI measures the volume of a region including white matter, whereas VBM gives a maximal specific point of change in grey matter only [4].

## Conclusion

It is evident that in populations at high risk of schizophrenia, transient or isolated psychotic symptoms occur relatively frequently. Sometimes these are precursors of schizophrenia but more often they are not. Cognitive and psychopathological features can be used to predict the development of schizophrenia [3], but they are more effective as negative than as positive predictors. In contrast, the changes in grey matter described here can be used as part of a positive predictive test for schizophrenia. This may be further improved in combination with other clinical and cognitive predictive measures [3], several of which are strong negative predictors (98% negative predictive value). Regardless, the results presented here indicate the possibility of early detection of schizophrenia with brain imaging in subjects at high risk for familial reasons. Although a sensitive issue, an accurate predictive test for (familial) schizophrenia could have substantial utility in assessing the possibilities for preventing the onset of this most disabling of disorders.

## List of abbreviations

SD: standard deviation

PSE: Present State Examination

VBM: voxel-based morphometry

ROC: receiver operator characteristic

ROI: region of interest

## Competing interests

The author(s) declare that they have no competing interests.

## Authors' contributions

ECJ, DGCO and SML were involved in the conception and design of the study and HCW, AMM, and DEJ, in the analysis and interpretation of data. All authors were involved in drafting the article or revising it critically for important intellectual content and read and approved the final manuscript.

## Pre-publication history

The pre-publication history for this paper can be accessed here:



## Figures and Tables

**Table 1:** Predictive statistics for schizophrenia using all 65 subjects.

Region	False+, False-	PPV%	NPV%	Likelihood ratio +	Likelihood ratio -	Sensitivity %	Specificity %	Effect size
Right cerebellum	7, 6	22	89	2.04	0.86	25	88	-0.82
Left uncus	4, 7	20	88	1.78	0.94	13	93	-0.33
Left inferior temporal gyrus	2, 5	60	92	**10.69**	0.65	38	96	-0.68

Statistics for the prediction of the development of schizophrenia within a 10-year period, based on changes in grey matter in the right cerebellum, left uncus and left inferior temporal gyrus (*n *= 65, all familial high-risk subjects with two scans in the first 4 years of the Edinburgh High Risk Study).

+ positive; - negative; NPV negative predictive value; PPV positive predictive value

**Table 2:** Predictive statistics for schizophrenia within those 18 subjects with minor symptoms.

Region	False+, False-	PPV%	NPV%	Likelihood ratio +	Likelihood ratio -	Sensitivity %	Specificity %	Effect size
Right cerebellum	2, 3	71	73	3.13	0.47	63	80	-0.68
Left uncus	3, 5	50	58	1.25	0.89	38	70	-0.09
Left inferior temporal gyrus	3, 3	63	70	2.08	0.54	63	70	-0.55

Statistics for the prediction of the development of schizophrenia within a 10-year period, based on changes in grey matter in the right cerebellum, left uncus and left inferior temporal gyrus (*n *= 18; only familial high-risk subjects who had transient, partial or isolated psychotic symptoms and had two scans in the first 4 years of the Edinburgh High Risk Study).

+ positive; - negative; NPV, negative predictive value; PPV, positive predictive value.
